# Influence of sampling design on landslide susceptibility modeling in lithologically heterogeneous areas

**DOI:** 10.1038/s41598-022-06257-w

**Published:** 2022-02-08

**Authors:** Andrei Dornik, Lucian Drăguţ, Takashi Oguchi, Yuichi Hayakawa, Mihai Micu

**Affiliations:** 1 Department of Geography, West University of Timisoara, Bd. V. Parvan 4, 300223 Timisoara, Romania; 2 Center for Spatial Information Science, The University of Tokyo, 5-1-5 Kashiwanoha, Kashiwa-shi, Chiba 277-8568 Japan; 3 Faculty of Environmental Earth Science, Hokkaido University, N10W5 Kita-ku, Sapporo, Hokkaido 060-0810 Japan; 4 Institute of Geography, Romanian Academy, 023993 Bucharest, Romania

**Keywords:** Environmental sciences, Natural hazards

## Abstract

This work aims at evaluating the sensitivity of landslide susceptibility mapping (LSM) to sampling design in lithologically-heterogeneous areas. We hypothesize that random sampling of the landslide absence data in such areas can be biased by statistical aggregation of the explanatory variables, which impact the model outputs. To test this hypothesis, we train a Random Forest (RF) model in two different domains, as follows: (1) in lithologically heterogeneous areas, and (2) in lithologically homogeneous domains of the respective areas. Two heterogeneous areas are selected in Japan (125 km^2^) and Romania (497 km^2^), based on existing landslide inventories that include 371 and 577 scarps, respectively. These areas are divided into two, respectively three domains, defined by lithological units that reflect relatively homogeneous topographies. Fourteen terrain attributes are derived from a 30 m SRTM digital elevation model and employed as explanatory variables. Results show that LSM is sensitive to a random sampling of the absence data in lithologically heterogeneous areas. Accuracy measures improve significantly when sampling and LSM are conducted in lithologically homogeneous domains, as compared to heterogeneous areas, reaching an increase of 9% in AUC and 17% in the Kappa index.

## Introduction

Landslides produce each year thousands of human victims^1^, important economic losses, as well as severe environmental damages. To reduce exposure to landslide hazards, it is necessary to identify the areas which are susceptible to such processes. Landslide susceptibility modeling (LSM) encompasses several branches of methods and approaches designed to evaluate the degree to which an area might be subject to landslide initiation^2^. Among these, statistically based methods have been increasingly used in the last four decades to perform quantitative LSM^2^. These methods describe the bivariate or multivariate statistical relationships between slope instability factors and the distribution of the inventoried landslides^3^, which are connected through spatial entities (raster grid cells or various types of vectors). Such entities are usually sampled randomly^4^ to train and validate the model and depending on the approach they can fall into two categories: landslide presence and landslide absence. Since sampling strategies hold a major influence on the results of the susceptibility models^5^, several studies have focused on this area^6^.

A category of sampling strategies has focused on presence data to address rather conceptual issues such as: how should landslides be modeled (single point vs. all points)?^7^; which parts of landslides should be considered (scarp vs. body)?^8^; which sampling strategy best approximates the pre-failure conditions? Other studies have considered both presence and absence data in sensitivity analyses of model performance related to sampling intensity (total or a relative number of samples) and the presence/absence ratio^9–11^. In such studies, presence data are usually sampled within landslides, and the absence samples are randomly selected from the areas outside the landslides^12^. Details on the above approaches, as well as references to them have been provided by Budimir et al.^13^, Lombardo et al.^14^, and Lombardo and Mai^15^.

Comparatively less attention has been paid to sampling of absence (non-landslide) data, although their quality is critical to the success of the models^16–18^. By analogy to ecological research, these data are rather pseudo-absence^19^ because their absence from landslide inventories does not necessarily mean that an area is stable^20^; it rather means that no evidence of landsliding has been found, either because of a biased survey (e.g. in forested areas), or because landslides have not occurred yet, so to be incorporated within an inventory^21^. The quality of the absence data depends more on the sampling strategy and less on the quality of the landslide inventory, as compared to the quality of the presence data^16^. Dhakal et al.^22^ proposed unaligned stratified random sampling (equal number of cells taken from rectangular blocks) to select the absence data and found that this method led to more accurate hazard (as called) maps as compared to a systematic sampling method. The success of this sampling strategy is likely due to the reduction of spatial autocorrelation as well as to the increase of the distance from the source area, which reduces confusions between presence and absence. Conoscenti et al.^23^ introduced a method to restrict sampling the absences to circles with diameters that approximate the width of the source area, which are distributed randomly over the apparently stable area. This approach also reduces the bias of the spatial autocorrelation, thus increasing the representativity and quality of the absence data. Later on, Conoscenti et al.^12^ showed that this sampling strategy helped in improving the accuracy of the models in the areas where the models were calibrated. However, the strategy was found less successful when the model was transferred outside the calibration area. Hong et al.^24^ evaluated the ratio of the presence to absence samples as a function of the size of the sampling area and found that the accuracy of LSM significantly depends on the interplay between the two factors. Shao et al.^25^ found that both sampling intensity and the presence/absence ratio impact the predicted occurrence probability of co-seismic landslides. Unlike the previous approaches, Zhu et al.^16^ proposed a sampling strategy that maximized the difference between presence and absence solely in the feature space. Their similarity-based sampling (SBS) approach led to better performances of the model as compared to other sampling strategies.

While the lithological and morphological homogeneity of the study area has been acknowledged as an important factor in the performance of LSMs^20,26^, most studies have not explicitly considered sampling issue. For instance, Blahut et al.^27^ performed LSM at a regional scale on both geomorphologically homogeneous zones and random partitions, and concluded that the former was preferable. Trigila et al.^28^ addressed the heterogeneity of the Italian territory by partitioning it into five more homogeneous domains for training LSMs. To facilitate an easier interpretation of the model by stakeholders, Petschko et al.^29^ fitted one generalized additive model (GAM) for each lithological unit in the study area. The authors reported a good ability of this approach to discriminate between slide and no slide points. Petschko et al.^30^ confirmed the merits of the partition on lithological units and mentioned that sampling bias can occur when some model domains have high contrast between so-considered stable (e.g. large flat areas) and unstable (e.g. steep areas) sectors. Similar results were reported by Steger and Glade^18^, who showed that in morphologically-heterogeneous regions, the presence of “trivial areas” (e.g. floodplains and flat areas) influenced modeled relationships, the appearance of landslide susceptibility maps, and associated prediction performance. Indeed, in heterogeneous areas some statistic trends in the explanatory variables may be different at the level of the individual domains as compared to their aggregate (the whole study area), thus leading to a fallacy in the models, which is known as the Simpson’s paradox. Such issues have been reported in geosciences, e.g. by Ma^31^ who noted that 3D geostatistical modeling can be plagued by sampling bias in heterogeneous areas. It should be noted that modeling on lithologically/morphologically homogeneous areas is not widely supported, because of the inherent subjectivity involved in the partition of the study area^32^.

Our analysis of the literature to date shows that the effects of sampling in heterogeneous areas are still poorly understood in LSM. Modeling in lithologically/morphologically homogeneous areas has been performed rather intuitively and/or for other purposes than understanding and improving the quality of sampling strategies. Moreover, it is not clear whether modeling in lithologically/morphologically homogeneous areas helps or just adds uncertainties through subjective delineation.

The main goal of this article is to evaluate the sensitivity of LSM to sampling in lithologically-heterogeneous areas. We hypothesize that random sampling of the landslide absence data in such areas can be biased by statistical aggregation of the explanatory variables, which impact the model outputs. To test this hypothesis, we train a Random Forest (RF) model in two different domains, as follows: (1) in lithologically heterogeneous areas, and (2) in lithologically homogeneous domains of the respective areas. The domains are defined by geological/lithological units that reflect more homogeneous topographies. The experiments are carried out in two different areas, considered representative of landslide-prone environments in Romania and Japan. Previous landslide susceptibility studies tended to deal with only one area or relatively close multiple areas. Therefore, the obtained results may strongly reflect specific local conditions. Here we selected two distant areas with clearly different environments, to obtain more widely applicable implications.

## Methods

### Study areas

**Figure 1:**
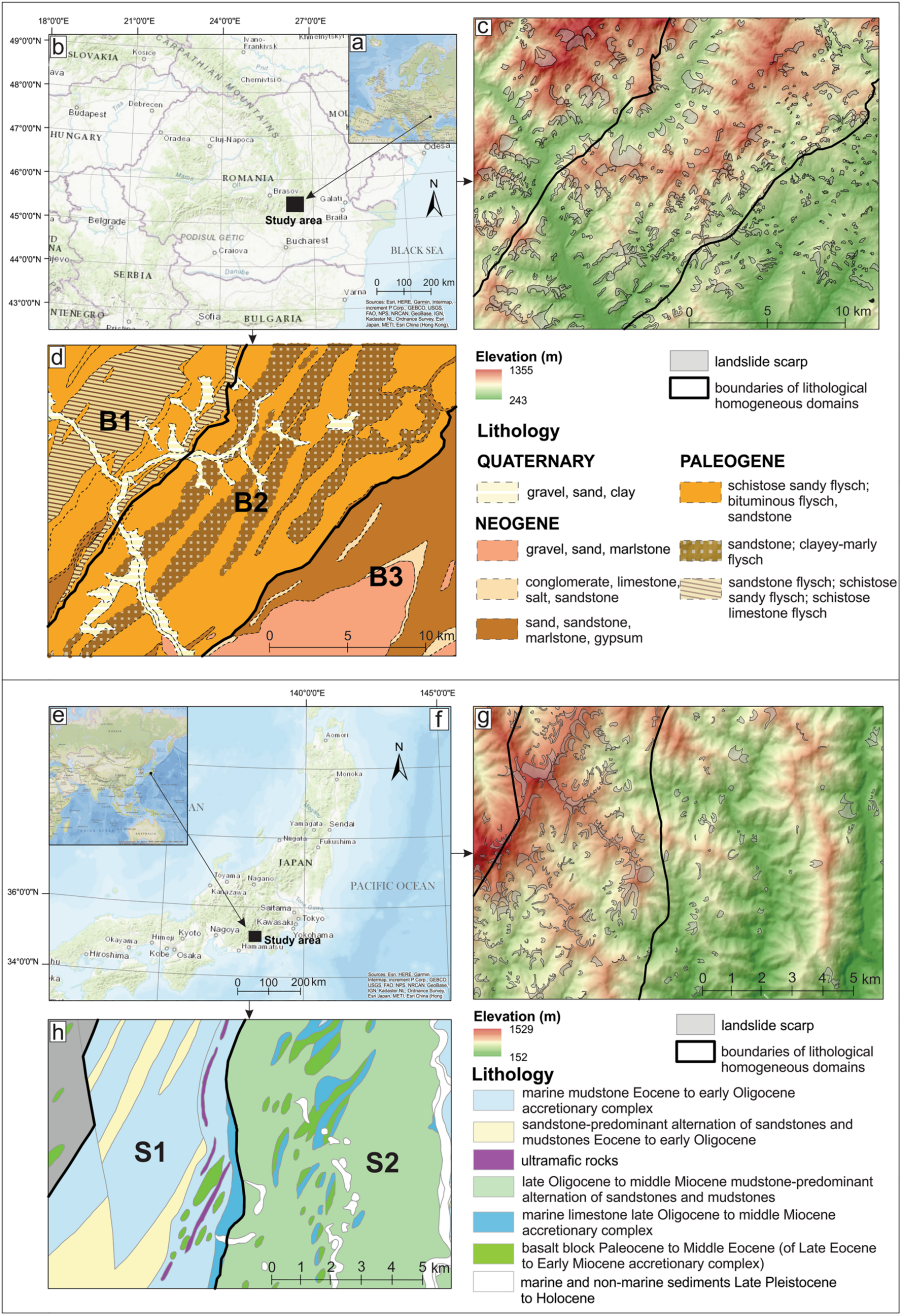
Location of Buzău (**a**,**b**) and Shizuoka (**e**,**f**) study areas. Lithologically-heterogeneous study areas are shown in (**c**) and (**g**) Lithologically-homogeneous domains are shown in (**d**) and (**h**).

The first study area is located within Buzău County, Romania, and covers about 497 km^2^, named hereafter B (Fig. 1a–c). It is a geologically and lithologically complex region due to complex morphogenesis and paleogeographic evolution in a tectonically active sector of an intra-collisional plates sector (Vrancea seismic region), developed at the contact between the Romanian Curvature Carpathian Mountains and the Subcarpathian Hills. In this area (generally well-covered by dense forests in the mountainous sector and by a mixed association of forests, orchards, and pastures/hayfields in the hilly sector) three distinct lithological domains could be individualized (Fig. 1d). The north-western section, named B1, belonging to the Carpathian Mountains (parts of Penteleu and Podul Calului sub-units), is built by low cohesive Paleogene flysch formations consisting of sandstones alternating with shale and schistose clayey-marly intercalations, and the maximum altitude of this area (1355 m) is the highest of the three domains. The central part covers a southwest-northeast-oriented structure, named B2, and is represented by a hilly massif reaching 1100 m (Ivănețu Ridge), which represents the external termination of the intensely folded Palaeogene flysch formations consisting of schistose sandy flysch with bituminous and clayey-marly intercalations. The third domain, named B3, is located in the south-east part of the study area and represents a part of the 600–800 m high Subcarpathian Hills (Buzău sub-unit), dominated by typical molasse deposits consisting of clays, marls, sand, and gravel deposits, with thin intercalations of schistose sandstone and gypsum (Fig. 1d).

The second study area is located in the southeast of Honshu Island, Japan, more precisely in the Shizuoka Prefecture, covering about 125 km^2^, named hereafter S (Fig. 1e–g). The study area is mainly covered by forests, reaching a maximum altitude of 1529 m. This area is subdivided into two domains, one located in the west (S1) and the other in the eastern part (S2). S1 has higher altitudes and consists mostly of the Eocene to early Oligocene marine sandstone and accretionary complex, with local patches of the sandstone-dominant alternation of sandstones and mudstones of the same age. S2 with lower altitudes is dominated by the late Oligocene to middle Miocene mudstone-predominant alternation of sandstones and mudstones, with local patches of marine limestone of the same age (Fig. 1h).

As in Petschko et al.^30^, the main criteria in the delineation of the domains was lithological homogeneity, as defined by similar geomechanical properties. However, delineation of each lithological unit would lead to excessively small areas, and some of them are without any landslide sample, which would make statistical modeling impossible. Thus, a tradeoff between “pure” homogeneity and the statistically relevant size of the domains had to apply. Therefore, smaller lithological units were merged into larger, relatively homogeneous associations, with similar geomechanical properties and visible expressions in topography (Fig. 1).

### Data

#### Landslide inventory database

In the Buzău study area**,** there is an available database containing 577 landslide scarps, acquired in the last 40 years and compiled from different sources, such as archive data, detailed geomorphological field mapping, local authority databases, digital stereographic photo interpretation using color aerial ortho-photographs^33,34^. For the Shizuoka study area, we used an inventory of 371 landslide scarps, provided by the National Research Institute for Earth Science and Disaster Resilience, Japan (NIED)^35–37^. The inventory was derived by visual interpretation of topographic discontinuities using stereo-paired aerial photographs at a 1:40,000 scale, acquired in the 1970s^33^. Both inventories lack attributes regarding landslide types.

The study areas and morphology of landslides as well as a more in-depth analysis of the existing databases are described in detail by Sîrbu et al.^33^.

#### Terrain variables

For both regions, we used the Shuttle Radar Topography Mission (SRTM) digital elevation model (DEM) with a spatial resolution of 30 m and an absolute vertical accuracy of the elevation data of 16 m. This product was created with radar interferometry technique^38^.

The System for Automated Geoscientific Analyses (SAGA) software was used with the DEM to derive 14 terrain variables, related to terrain slope and curvature, landscape position, and terrain roughness. Elevation is regularly used as a predictor in landslide susceptibility assessment^39^, while slope gradient is the most widely used predictor in landslide modeling^2^. Slope aspect may influence the soil moisture and vegetation growth^3^. In this study, the slope aspect has been expressed as cosine and sine of the aspect, which represents the northerness and easterness, respectively. Slope length and slope height are related to potential disruption energy^40^. Plan curvature is generally used to highlight the divergence or convergence of flow, while profile curvature and convexity describe the relative deceleration/acceleration of material flows^40^. Valley depth and midslope position illustrates the relative landscape position, acknowledged as a predictor of landslides^41^. In addition, landscape position, related to dominance or enclosure of a location, is illustrated by topographic positive openness, and negative openness respectively^42^. Terrain surface texture, emphasizing differences in elevation of different locations, is related to terrain roughness^43^.

### Sampling strategy

In each lithologically heterogeneous area (B and S) and lithologically homogeneous domain (B1, B2, B3, S1, and S2) we conducted a random sampling. From all the available points (all pixels) in each area, we have selected a representative number of points, using the *r.sample* command^44^ within the Geospatial Modelling Environment^45^, ensuring 50% within landslide scarps (at least one point per scarp) and 50% outside landslide scarps. Representative numbers of samples were calculated for a margin of error of 1% and a confidence level of 95%. For the Buzău area, the representative number is 10,000, while in Shizuoka it is 6000.

The seven point-type databases were subsequently intersected with the stack of terrain variables, recording the following information: presence/absence of landslide scarp and associated values of the terrain variables.

### Random Forest models

The random forest (RF) method was used for conducting LSM. RF^46^ is a machine-learning algorithm for non-parametric multivariate classification or regression, being increasingly used in environmental modelling^47^.

The randomForest package in R software^44^ was applied on the seven point-type databases. The predictors consisted of all 14 terrain variables, and LSM was conducted separately within each lithologically heterogeneous area (B and S), as well as in each lithologically homogeneous domain (B1, B2, B3, S1, and S2), resulting in seven models and susceptibility maps.

In addition, to evaluate confidence limits and uncertainties, a bootstrapping technique was used by repeating the random sampling and LSM 20 times. To create the LSM map for each area, the RF model with the maximum accuracy was retained among all models. To assess independently the accuracy of models, at each iteration the database was split randomly into two parts: 70% of samples were used for LSM prediction, while the rest 30% were used for model evaluation.

### Model evaluation

To test the hypothesis, results are compared both quantitatively as model prediction performance and relative to their geomorphic plausibility. The prediction performance of the LSM models was assessed by four metrics. The first metric is the area under the curve (AUC), a widely used metric for model performance evaluation^2^ and is also the most appropriate method for assessing binary classification. AUC is also easy to interpret, since it has a value of 0.5 for a test with accuracy no better than chance, while a value of 1 for a test with perfect accuracy^48^. The second metric is “out-of-bag error” (OOB), an estimator of the ensemble error, computed by comparing the out-of-bag predicted responses against the true responses^39^. The other metrics are overall accuracy (OA) and the kappa index of agreement (Kappa), widely known and used in environmental mapping. OA and Kappa have been used to assess the predicted classes (scarp vs no scarp), with Kappa compensating for the success of a random classification. They were used since OOB may overestimate the true prediction error because of binary classification problems. AUC was used to evaluate the landslide susceptibility models (probability maps) and measures how well the classifier ranks the probability of scarp class higher than no scarp class. The values of accuracy metrics were retained for all repetitions to assess the variability and confidence limits of the LSM.

## Results

**Figure 2:**
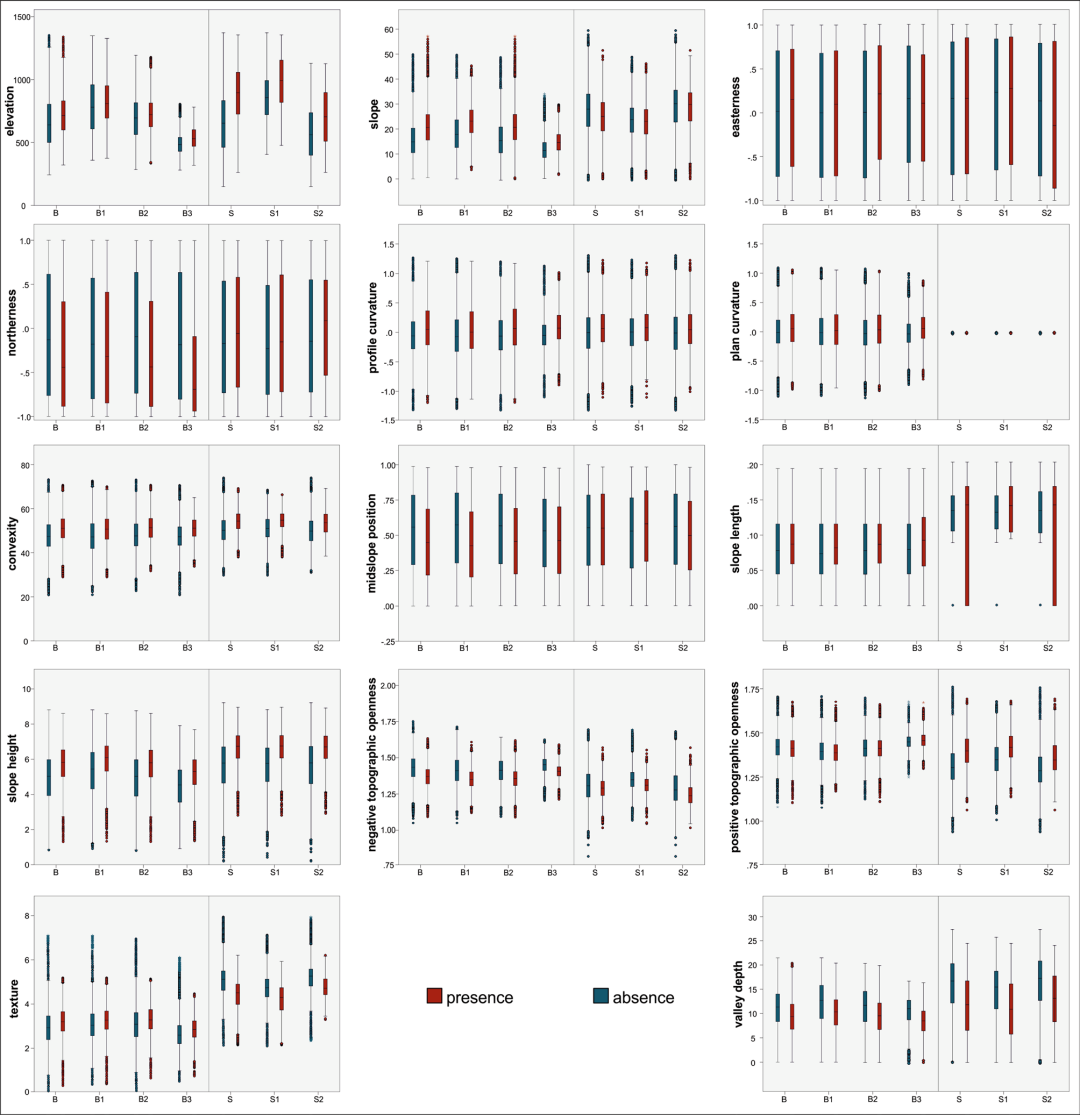
Distribution of terrain variables in the landslide presence and absence areas as represented by the box plot. Note that those variables with non-normal distributions have been subjected to normalization procedure.

The distribution of elevation values in the entire population of the Buzău area shows a notable decrease of variation within homogeneous lithological domains as compared to the entire heterogeneous area, except for B1 (Fig. 2). The elevation of absence data within the study area B has an interquartile range of 303 m (1st quartile 501 m; 3rd quartile 804 m) and a median of 640 m, while B2 has a range of 252 m (1st quartile 565 m; 3rd quartile 817 m) with the median of 697 m, and B3 has an interquartile range of 110 m (1st quartile 432 m; 3rd quartile 542 m) with a median value of 532 m. Similarly, the interquartile range of elevation among presence data is significantly lower in B2 (188 m) and B3 (131 m) as compared to the heterogeneous area B (231 m). B1 is the only homogeneous domain that recorded a higher interquartile range compared to B, with values of 351 m for absence and 257 m for presence (Fig. 2).

In the Shizuoka study area, only absence data recorded an interquartile range significantly lower within homogeneous domains; S1 with 272 m (1st quartile 722 m; 3rd quartile 994 m) and S2 with 337 m (1st quartile 401 m; 3rd quartile 738 m), compared to S with an interquartile range of 370 m (1st quartile 464 m; 3rd quartile 834 m). The presence data recorded a similar interquartile range in S1 (336 m) as compared to S (332 m), but a higher value in S2 (386 m) (Fig. 2).

In both study areas, aggregation leads to an increase in the differences between presences and absences, which is more visible in the Japanese case (Fig. 2). In Buzău, the difference in the median values between presences and absences decreases from 75 m in B to 28 m in B1, 25 m in B2, and 46 m in B3. In Japan, the difference is 241 m for S, 136 m for S1, and 143 m for S2.

**Figure 3:**
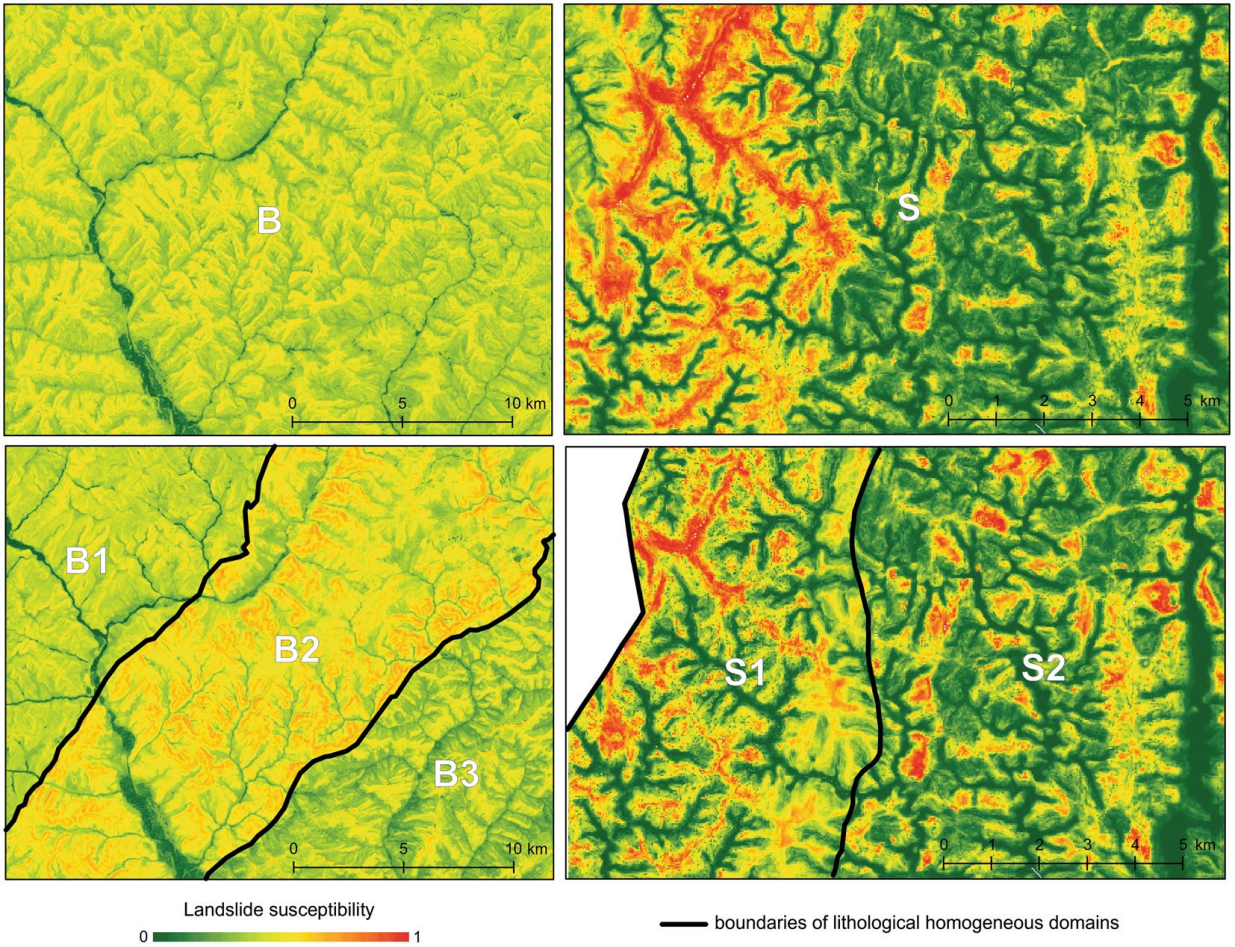
Landslide susceptibility maps in Buzău (left) and Shizuoka (right).

Landslide susceptibility maps in the Buzău and Shizuoka study areas, both within the heterogeneous lithological area (B and S) and homogeneous domains (B1, B2, B3, S1, S2) are shown in Fig. 3. Different spatial distributions of the values can be observed between mapping within a heterogeneous lithological area and homogeneous domains, although the susceptibility values are similar and range from 0.017 to 0.655 in B, from 0.001 to 0.637 in B1, from 0.018 to 0.775 in B2, and from 0.06 to 0.698 in B3, respectively, while in Shizuoka they range from 0 to 0.99–1.

By using a representative number of samples, a stratified random sampling led to an increase of AUC by 7% in B1, 1% in B2, and 9% in B3 as compared to the heterogeneous area, in all three lithological homogeneous areas in Buzău. The other three metrics show a similar trend of performance increase when sampling is conducted within lithological homogeneous domains, except the kappa index in B2 (Table 1). The S1 area is the only case study out of five, in which the stratified random sampling resulted in poorer prediction as compared to the heterogeneous area, with AUC recording a lower value by 4%. In S2, all four metrics show significant improvement of stratified sampling over random sampling in the heterogeneous area, by 3% for AUC, 6% for OOB, 5% for OA, and 9% for Kappa, respectively (Table 1).

**Table 1:** Accuracy metrics with a representative number of samples.

Study area	Number of samples	One random sampling				20 repetitions			
		OOB	AUC	OA	Kappa	OOB	AUC	OA	Kappa
B	10,000	0.26	0.82	0.75	0.51	0.26	0.82	0.75	0.49
B1	10,000	0.19	0.89	0.80	0.61	0.20	0.90	0.81	0.62
B2	10,000	0.25	0.83	0.75	0.49	0.26	0.83	0.75	0.50
B3	10,000	0.18	0.91	0.83	0.65	0.18	0.91	0.83	0.66
S	6000	0.20	0.91	0.82	0.65	0.19	0.89	0.81	0.63
S1	6000	0.23	0.87	0.78	0.56	0.23	0.87	0.79	0.57
S2	6000	0.14	0.94	0.87	0.74	0.14	0.94	0.87	0.73

*OOB* out-of-bag error, *AUC* area under the curve, *OA* overall accuracy, *Kappa* kappa index of agreement.

**Figure 4:**
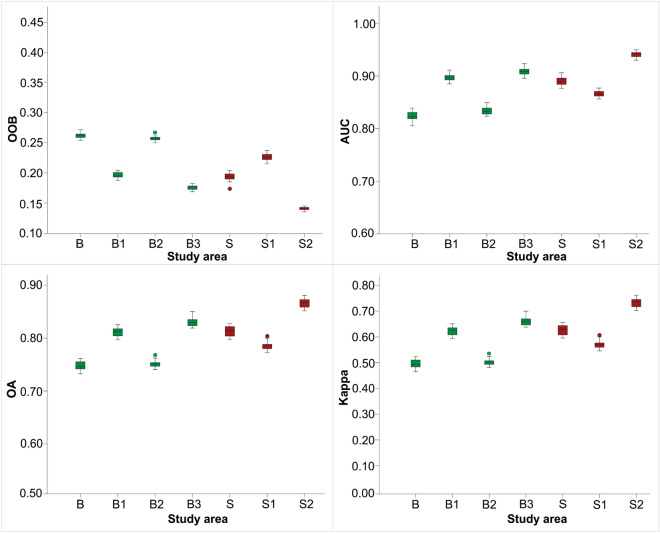
Distribution and variation of accuracy metrics after 20 repetitions. The Buzău study areas are shown in green; the Shizuoka study areas are shown in red. *OOB* out-of-bag error, *AUC* area under the curve, *OA* overall accuracy, *Kappa* kappa index of agreement.

The differences between single sampling and an average resulting from 20 times repetitions are insignificant, by only 1–2% (Table 1), which suggests high confidence of LSM even when only one draw is performed. Also, the variation of the four-accuracy metrics among 20 repetitions is small, up to 1–2%, within all study areas (Fig. 4).

## Discussion

To evaluate the sensitivity of LSM to sampling in heterogeneous areas, we performed LSM in two study areas, on the whole regions (B and S) as well as at the level of their lithologically homogeneous domains (B1, B2, B3, S1, S2) and found that the accuracy of modeling increased when the sampling was restricted to the homogeneous domains, except for one case (S1). These results are explained by differences in the statistical properties of the explanatory variables associated with the landslide absence data. As lithology is often reflected in topography, terrain variables tend to vary accordingly, so that their statistical properties might differ when computed at the level of lithological strata versus larger, more heterogeneous areas. For instance, altitude values exhibit more variation, as well as higher differences between presences and absences at the level of entire study areas (B and S) as compared to their homogeneous domains (Fig. 2). A random sampling of absence data brings this exaggerated difference into the prediction model, which associates the presence with high altitudes and the absence with low heights, respectively. Thus, the scarps in the lower areas are underestimated, while those in the higher parts of the landscape are overestimated, as visible in Fig. 3. This bias also occurs in S1 where the low-altitude scarps present in the southern part of the area (Fig. 1), on a different lithology, are underestimated. Such differences in global vs. local are present in all other terrain variables and propagate errors in the prediction models.

**Figure 5:**
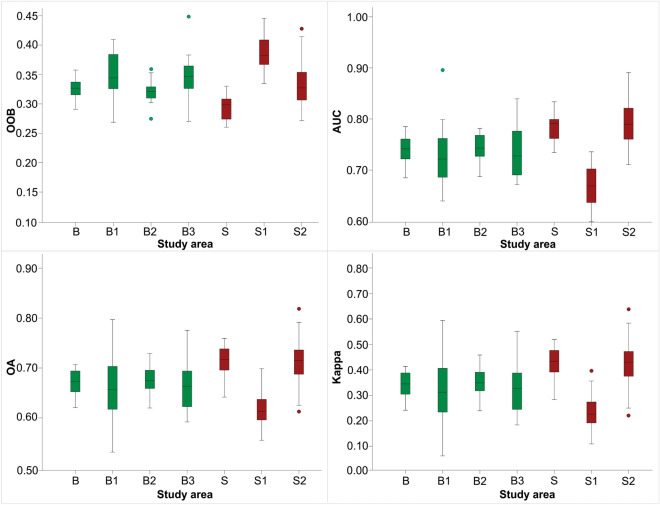
Distribution and variation of accuracy metrics after 20 repetitions, with one point per scarp. The Buzău study areas are shown in green; the Shizuoka study areas are shown in red. Additional explanations are found in Fig. 4.

Similar improvements in the accuracy of the results on the lithologically homogeneous areas were also observed when applying the widely used sampling strategy of one point per scarp and a balanced number of absences^49^. Thus, we have sampled one random point within each landslide scarp polygon and the same number of points randomly created outside the landslide scarp area, as absence data, both in the heterogeneous areas (B and S) and their homogeneous domains (B1, B2, B3, S1, S2). The accuracy metrics are generally higher for the models run on the lithologically-homogeneous domains (Table 2). However, the values of accuracy measures are lower as compared to the analysis based on the representative number of samples. On the other hand, the use of the strategy of one point per scarp leads to unstable results. By repeating the sampling 20 times, we obtained accuracy metrics that exhibit large interquartile ranges, with total and partial overlapping within the Buzău study areas (Fig. 5).

**Table 2:** Accuracy metrics with one point per scarp.

Study area	Number of samples	One random sampling				20 repetitions			
		OOB	AUC	OA	Kappa	OOB	AUC	OA	Kappa
B	1154	0.30	0.77	0.73	0.45	0.33	0.74	0.67	0.34
B1	204	0.26	0.81	0.77	0.54	0.35	0.73	0.66	0.32
B2	220	0.36	0.74	0.70	0.39	0.32	0.74	0.67	0.35
B3	220	0.30	0.78	0.75	0.50	0.35	0.73	0.67	0.34
S	714	0.29	0.78	0.72	0.44	0.29	0.78	0.71	0.43
S1	220	0.32	0.84	0.75	0.50	0.39	0.67	0.62	0.23
S2	220	0.29	0.82	0.77	0.54	0.34	0.79	0.72	0.43

*OOB* out-of-bag error, *AUC* area under the curve, *OA* overall accuracy, *Kappa* kappa index of agreement.

The results reported here confirm the findings by Blahut et al.^27^ and Petschko et al.^29^ that LSM is more accurate when conducted on lithologically/morphologically homogeneous areas. Spatial modeling in heterogeneous areas has been acknowledged as an important issue in Geoinformatics because spatial heterogeneity leads to uncontrolled variance in geographic variables^50^. We showed that random sampling of the absence data significantly influences the accuracy of LSM because of the differences in the statistical properties of the explanatory variables at homogeneous vs. heterogeneous levels. This sampling bias did not display reversals of statistical relationships at the heterogeneous levels as in a typical Simpson’s paradox. Nevertheless, a sampling bias that does not lead to a reversal can be more dangerous as it is less noticeable, thus holds a higher potential for biased results^31^.

On the other hand, our results disagree with the sampling strategy of one point per scarp and a balanced number of absences. By following such a strategy, the accuracy metrics were highly variable, especially in the Buzău study area, where the results were likely obtained by chance. Recently, Shao et al.^25^ found that varying the sampling intensity led to significant differences in the total predicted landslide area and concluded that stability of the prediction results increases with the intensity of the sampling. In contrast, we obtained acceptable results with relatively low interquartile ranges of accuracy metrics, by selecting a representative number of samples, calculated for a margin of error of 1% and a confidence level of 95%. A recent study^8^ also found that the predictive power of the models generally increases with sampling intensity, yet the differences in accuracy are canceled out by using a deep learning neural network as compared to other models.

Seen from a slightly different perspective, our results can be explained by the interpretation of lithology as a lurking variable. According to this view, lithology was ignored in the heterogeneous areas (B and S), while implicitly considered in modeling their homogeneous domains (B1, B2, B3, S1, S2). In other words, lithology is important in LSM, thus it should be added as an explanatory variable in modeling. However, a meaningful coding of lithology as a variable is not easy, because the information included within the geological maps may not have a direct relationship with the mechanical properties of the rocks^2^. Perhaps this difficulty is the reason for its scarce use in statistically-based LSM—less than 10%, according to a recent review^2^. Stratification of a landscape according to lithology is, in turn, more straightforward when considering only the lithological boundaries that mark topographic discontinuities as well.

In this study, we did not consider the landslide types because this information was not available in the inventories. Since it is known that the accuracy of predictions improves when the classification of landslide type is made first^4^, we expect better results from such an approach at least for some types of landslides, as shown by Shu et al.^51^.

## Conclusions

This study has shown that landslide susceptibility mapping is sensitive to a random sampling of the absence data in lithologically/morphologically heterogeneous areas. Accuracy measures improved significantly when sampling and LSM were conducted in lithologically homogeneous domains, as compared to heterogeneous areas, reaching a maximum increase of 9% for AUC and 17% for the Kappa index. These results are explained by differences in the statistical properties of the explanatory variables associated with the landslide absence data, which impact the model outputs.

We also found that the results of LSM are more stable when selecting a representative number of samples, calculated for a margin of error of 1% and a confidence level of 95%. Repetition of the random sampling with this design 20 times resulted in low differences in accuracy measures. By contrast, the sampling strategy of one point per scarp and a balanced number of absences led to large interquartile ranges of accuracy metrics, with the results probably obtained by chance, which is obvious in one study area.
